# Lycorine Inhibits Hypertrophic Scar Formation by Inducing ROS-Mediated Apoptosis

**DOI:** 10.3389/fbioe.2022.892015

**Published:** 2022-05-24

**Authors:** Yunxian Dong, Dongming Lv, Zirui Zhao, Zhongye Xu, Zhicheng Hu, Bing Tang

**Affiliations:** ^1^ Department of Burn and Plastic Surgery, The First Affiliated Hospital of Sun Yat-sen University, Guangzhou, China; ^2^ Department of Gastrointestinal Surgery, The First Affiliated Hospital of Sun Yat-sen University, Guangzhou, China

**Keywords:** hypertrophic scar, lycorine, apoptosis, reactive oxygen species, fibrosis

## Abstract

**Background:** Hypertrophic scar (HS) is a fibrotic cutaneous disease with few effective therapies. Lycorine is a drug with pro-apoptotic ability and anti-fibrosis potential. This study aimed to test whether lycorine could trigger the apoptosis of hypertrophic scar fibroblasts (HSFs) to inhibit HS formation.

**Methods:** The proapoptotic and anti-fibrosis effects of lycorine on the viability and apoptosis of human primary HSFs and their reactive oxygen species (ROS) production as well as a rabbit ear model of HS were determined by CCK-8, flow cytometry, Western blot, immunofluorescence, transwell migration, collagen gel contraction assays.

**Results:** Lycorine treatment selectively decreased the viability of HSFs, and induced their apoptosis, but not normal fibroblasts (NFs). Lycorine treatment increased the relative levels of Bax and cleaved PARP expression, cytochrome C cytoplasm translocation, but decreased Bcl-2, caspase-3 and caspase-9 expression, the mitochondrial membrane potential (MMP) in HSFs. Lycorine inhibited the migration and contraction of HSFs, and reduced the expression of collagen I, collagen III and α-SMA. Mechanistically, lycorine treatment stimulated high levels of ROS production, leading to apoptosis of HSFs while treatment with NAC, a ROS inhibitor, significantly mitigated or abrogated the pro-apoptotic and antifibrotic activity of lycorine in HSFs. Moreover, lycorine treatment mitigated the severity of HS in rabbit ears by inducing fibroblast apoptosis.

**Conclusion:** These results indicate that lycorine has a potent anti-fibrotic activity and is a potential drug for intervention of HS.

## Introduction

Hypertrophic scar (HS) is a fibrotic disease and particularly affects the appearance and function of the skin (Finnerty et al., 2016). Generally, various cutaneous injuries, such as burns, trauma and skin surgery, may cause pathological scarring of the dermis (Limandjaja et al., 2021). At present, non-surgical treatment for HS includes laser therapy, compression therapy, silicone, injection with corticosteroids, however, these therapeutic strategies are limited (Lee and Jang, 2018; Park et al., 2019; Manuskiatti et al., 2021). Hence, the development of new anti-scar therapies is necessary.

Mechanistically, HS is mainly caused by two factors, the continuous proliferation of dermal myofibroblasts and excessive deposition of extracellular matrix (ECM), such as collagen fibers in scar tissue (Finnerty et al., 2016). After skin injury, many proliferating fibroblasts participate in tissue repair and secrete high levels of α-smooth muscle actin (α-SMA) and collagens, transforming to the myofibroblast phenotype (Shin and Minn, 2004). Of note, α-SMA can enhance the contraction ability of HSFs, while collagen accumulation in ECM can lead to scar hardening, protruding skin surface, and the joint scar can cause limb contracture deformity (Kwan and Tredget, 2017). Therefore, the inhibition of myofibroblast formation and decrease in fibrogenic factors, such as collagen, α-SMA, will be important to limit scar formation following a skin wounding.

The proliferating HSFs usually ignore apoptosis triggers. Accordingly, selective triggering HSF apoptosis may be a valuable strategy for intervention of HS (Feng et al., 2020; Zhu et al., 2020). Lycorine is a kind of natural alkaloid extracted from medicinal plants in the lycoris family (Cao et al., 2013). Previous studies have shown that lycorine can inhibit the proliferation of various types of cancer cells (Lamoral-Theys et al., 2009; Roy et al., 2018) and induce the apoptosis of human hepatoblastoma HepG2 and breast cancer MCF-7 cells (Ji et al., 2017; Liu et al., 2019). In addition, lycorine has been used as a novel natural compound for the treatment of cardiac fibrosis (Schimmel et al., 2020) and alleviates the bleomycin-induced pulmonary fibrosis in rodents (Liang et al., 2020). However, there is no information on the roles of lycorine in skin fibrosis.

Based on its proapoptotic ability and anti-fibrosis effect, this study tested the hypothesis that treatment with lycorine could ameliorate HS in human primary skin fibroblasts and a rabbit model of HS.

## Materials and Methods

### Cell Culture

The skin tissues were obtained from 17 volunteers, including 12 HS patients (6 female, 6 male, age 20–39 years), who were diagnosed pathologically, and five non-HS subjects (three males and two females, ages 18–29 years). Briefly, the dermal portion of scar tissues or normal skin tissues were cut into small pieces (1 mm^3^) and digested with collagenase type II (S10054, 0.8 mg/ml, Yuanye, China) at 37°C for 6–8 h (Shi et al., 2017). The generating HS fibroblasts (HSFs) and normal fibroblasts (NFs) were cultured in DMEM medium (Gbico, United States) supplemented with 10% fetal bovine serum (FBS, Gbico) at 37°C in 5% CO_2_. The cells at passage 3–5 were used for following experiments.

### Cell Viability Assay

HSFs and NFs (2 × 10^3^cells/well) were cultured in 96-well plates overnight and treated in triplicate with different doses (0, 2.5, 5.0, 7.5, 10.0, 20.0, 30.0, 40.0 μmol/L) of lycorine (Glpbio, United States, GC38967) in dimethyl sulfoxide (DMSO, Sigma, United States) for 12–48 h. The cell viability was determined using a CCK8 kit (Fdbio, China, FD3788) per the manufacturer’s protocol.

### Flow Cytometry Analysis of Cell Apoptosis

HSFs and NFs were cultured in 6-well plates overnight and treated in triplicate with vehicle or lycorine at 10, 20, and 40 μmol/L for 24 h. The cells were harvested and stained with Annexin V-FITC and propidium iodide (PI, 4abio, China) in the dark. After being washed, the frequency of apoptotic cells was analyzed by flow cytometry.

### Western Blot Assay

HSFs were treated with vehicle or lycorine (10, 20, 40 μmol/L) for 24 h, harvested and lyzed in radioimmunoprecipitation assay (RIPA, Fdbio, FD008) buffer containing inhibitors of protease (Fdbio, FD1001) and phenylmethylsulfonyl fluoride (PMSF, Fdbio, China, FD0100), followed by centrifugation. The concentrations of lysate proteins were quantified using the BCA protein assay kit (Fdbio, FD 2001). The cell lysate samples (50 µg/lane) were separated by SDS-PAGE on 7.5%–12.5% gels and transferred to polyvinylidene difluoride (PVDF) membranes (Millipore, Temecula, CA, United States). After being blocked with 5% non-fat dry milk in TBST buffer for 1 h, the membranes were incubated with primary antibodies at 4°C overnight. The bound antibodies were reacted with horseradish peroxidase (HRP)-conjugated secondary antibodies (Cell Signaling Technology, #3108, #7076, 1:5000). Similarly, the collected ear tissues were homogenized and subjected to Western blot analysis. Mitochondrial proteins were extracted from each group of cells or tissues using the Cell Mitochondria Isolation Kit (Beyotime, China, C3601).

The primary antibodies were used at 1:1000 dilution, including Bax (CST, United States, #5023S), Bcl-2 (CST, #15071S), caspase9 (Proteintech, United States, 10380-1-AP), caspase3 (Proteintech, 19677-1-AP), PARP (Proteintech, 13371-1-AP), c-PARP (CST, #5625), anti-Cyt-c (Proteintech, 10993-1-AP), GAPDH (60004-1-Ig, 1:5000), anti-Cox-IV (CST, #4850), Collagen I (Proteintech, 14695-1-AP), Collagen III (Proteintech, 22734-1-AP), α-SMA (Proteintech, 14395-1-AP).

### Mitochondrial Membrane Potential Assay

The impact of lycorine on mitochondrial membrane potential of HSFs was analyzed using JC1 (MCE, HY-15534, United States) staining. Briefly, HSFs were treated in triplicate with different doses of lycorine at 37°C for 24 or 48 h and stained with JC-1 for 15 min at 37°C in the dark. After being washed, the cells were analyzed under a fluorescence microscope (LSM880 Basic Operation, Zeiss, Germany).

### Transwell Migration Assay

The HSFs were harvested and HSFs at 2 × 10^4^ cells/well were cultured in FBS-free medium containing vehicle or 10, 20, and 40 μmol/L of lycorine in the upper chamber of 24-transwell plate. The bottom chambers were filled with 600 µl of medium containing 20% FBS. After being cultured for 24 h, the cells on the up-surface of chamber were removed using a cotton ball and the cells that had migrated onto the bottom surface of the chamber were fixed with paraformaldehyde for 30 min and stained with 0.1% crystal violet for another 30 min. The numbers of migrated cells in five visual fields ( × 100 magnification) were counted under a microscope in a blinded manner.

### Collagen Gel Contraction Assay

HSFs (5 × 10^5^ cells/mL, 2 ml) were mixed with rat tail collagen (900 μl, 5 mg/ml, Solarbi, China, C8062) and 10 × DMEM (100 μL) (pH ≈ 7) and cultured in 24-well plates (1 ml/well) in the presence of lycorine (10, 20, 40 μmol/L) or vehicle at 37°C for 30–60 min. The resulting collagen gel in each well was taken out and photoimaged at 12 or 48 h later. The changes in gel areas were calculated.

### Immunofluorescence Staining

HSFs were cultured on glass coverslips in dishes overnight, and treated with different concentrations of lycorine for 24 h. The cells were fixed with 4% paraformaldehyde for 30 min, permeabilized with 0.25% of Triton-×100 for 20 min and blocked with goat serum for 1 h. The cells were incubated with primary antibodies overnight at 4°C. After being washed, the bound antibodies were reacted with fluorescent secondary antibodies at room temperature for 1 h and co-stained with DAPI. The fluorescent signals were captured under a confocal microscope (LSM880 Basic Operation, Zeiss, Germany). Similarly, rabbit ear scar tissue sections were fixed in 4% paraformaldehyde and subjected to immunofluorescence. The primary antibodies included Collagen I (Proteintech, 14695-1-AP), Collagen III (Proteintech, 22734-1-AP), caspase9 (Proteintech, 10380-1-AP).

### Analysis of Reactive Oxygen Species

The impact of lycorine on ROS production in HSFs was determined by flow cytometry and immunofluorescence. Briefly, HSFs were treated in triplicate with vehicle or lycorine at 37°C for 24 h and after being washed, the cells were cultured in serum-free DMEM in the presence of 10 µM 2,7-dichlorofluorescein diacetate (DCFH-DA,APExBIO, Houston, TX, United States, C3890) at 37°C for 30 min in the dark. The cells were harvested for flow cytometry analysis in a Beckman Cyan flow cytometer (Beckman Coulter, Brea, CA, United States) and immunofluorescence analysis.

### Real-Time Quantitative PCR

Total RNAs were extracted from scar tissue samples using RNA Easy Fast Tissue/Cell kit (TIANGEN, China) and reversely transcribed into cDNA using the Thermo Scientific RevertAid First Strand cDNA Synthesis Kit (Thermo Scientific, Waltham, MA, United States), according to the manufacturer s instructions. The relative levels of interesting gene mRNA transcripts to the control GAPDH were quantified by RT-qPCR using specific primers and SYBR Green PCR master mix (Toyobo, Osaka, Japan) on a Bio-Rad CFX96 Real Time PCR system (Bio-Rad, Hercules, CA, United States). The data were analyzed by 2^−ΔΔCt^. The sequences of primers were used, according to a previous study (Chen et al., 2018):

Bcl-2-F: 5’ -GAT​TGT​GGC​CTT​CTT​TGA​GTT​C-3′
Bcl-2-R: 5’ -AAG​TCT​TCA​GAG​ACA​CCC​AGG​A-3′
Bax-F: 5’ -TTT​GCT​TCA​GGG​TTT​CAT​CC-3′
Bax-R: 5’ -GGC​AGC​GAT​CAT​CCT​CTG​TA-3′
GAPDH-F: 5’ -GAA​TCC​ACT​GGC​GTC​TTC​AC-3′
GAPDH-R: 5’ -CGT​TGC​TGA​CAA​TCT​TGA​GAG​A-3′

### A Rabbit Model of Ear Hypertrophic Scar

New Zealand white rabbits, weighing 2.0–2.5kg, were from Guangdong Medical Laboratory Animal Center (approval number: SCXK (yue) 2019–0035). All animal experimental protocols were approved by the Animal Experimental Center of the First Affiliated Hospital of Sun Yat-sen University. To establish a rabbit model of ear HS, the rabbits were anesthetized and the ventral surface of each ear was wounded with four 10-mm cylindrical cores (full-thickness and the perichondrium was preserved) using a 10-mm biopsy punch. After 14 days, the skin wound healed and scar was formed gradually. The rabbits were randomized and injected with 0.1 ml of vehicle or lycorine (5 mg/ml) into their ear scar region every 3 days (Zheng et al., 2021). The total duration of lycorine therapy was 14 days. At the end of the treatment (28 days post wounding), their scar tissues were dissected for histology, RT-qPCR and immunofluorescence analyses.

### Histopathology

The dissected ear tissues were fixed in 10% of formalin overnight and paraffin-embedded. The tissue sections (5 µM) were regularly stained with hematoxylin and eosin or immunohistochemistry (IHC) staining. The stained tissue sections were photoimaged and observed under a light microscope. The primary antibodies were used at 1:200 for α-SMA (Proteintech, 14395-1-AP), 1:400 for Collagen I (Proteintech, 14695-1-AP), Collagen III (Proteintech, 22734-1-AP).

### Statistical Analysis

The data are expressed as mean ± SEM. The difference between two groups was analyzed by the unpaired t-test analysis and the difference among multiple groups was analyzed by one-way ANOVA and post hoc Bonferroni’s correction using SPSS 24.0 software. A *p*-value of < 0.05 was considered statistically significant.

## Results

### Lycorine Decreases the Viability of Hypertrophic Scar Fibroblasts

**FIGURE 1:**
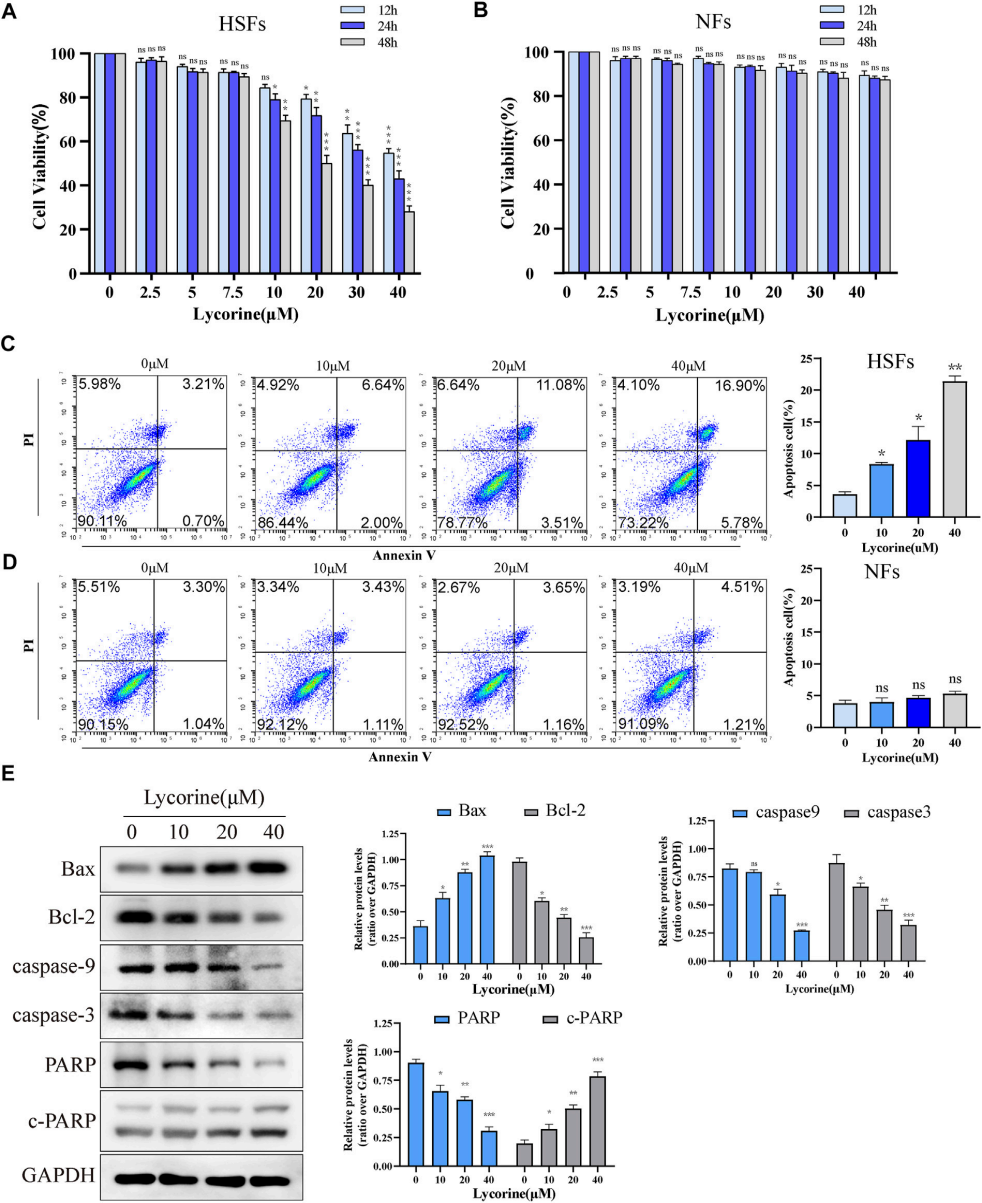
Lycorine reduces the viability of HSFs by triggering their apoptosis. HSFs and NFs were treated with vehicle or lycorine at indicated concentrations. At 12, 24 and 48 h later, cell viability (*n* = 5) of HSFs **(A)** and NFs **(B)** were determined by CCK-8 assay. Flow cytometric analysis of the frequency of apoptotic HSFs **(C)** and NFs **(D)** at 24 h post lycorine treatment (*n* = 4). **(E)** At 24 h post lycorine treatment, the relative levels of Bax, Bcl-2, caspase 9, caspase3, PARP and cleaved PARP (c-PARP) in HSFs were examined by Western blot (*n* = 3). Data are mean ± standard error of the mean. *p < 0.05, **p < 0.01, ***p < 0.001.

To examine the direct effect of lycorine on cells viability, human NFs and HSFs were treated with lycorine at different concentrations (0, 10, 20, 40 µM) up to 48 h and their viabilities were determined by CCK-8 assays. As shown in Figure 1A, treatment with lycorine from 10 to 40 µM significantly decreased the viability of HSFs in a dose-dependent manner. Treatment with 20 µM lycorine for 48 h reduced the viability by>50%, similar to that of treatment with 40 µM lycorine for 24 h. The half maximal inhibitory concentration (IC50) of lycorine for HSFs with different duration were 43.63 µM (12 h), 34.54 µM (24 h), 25.16 µM (48 h), respectively. However, treatment with lycorine at the same dose ranges did not significantly alter the viability of NFs (Figure 1B). Hence, lycorine treatment selectively decreased the viability of HSFs in a dose and time-dependent manner.

### Lycorine Increases the Intrinsic Apoptosis of Hypertrophic Scar Fibroblasts

Next, the effect of lycorine on fibroblast apoptosis was tested. NFs and HSFs were treated with different concentrations of lycorine for 24 h, the frequency of apoptotic cells was determined by flow cytometry (Figure 1C). Treatment with different doses of lycorine increased the percentages of apoptotic HSFs in a dose-dependent manner (Figure 1C). However, the same treatments did not significantly increase the percentages of apoptotic NFs in our experimental conditions (Figure 1D). Furthermore, Western blot uncovered that treatment with varying doses of lycorine significantly increased the relative levels of Bax, cleaved poly ADP-ribose polymerase (c-PARP), but gradually decreased Bcl-2, caspse-9 and caspase-3 as well as PARP expression in HSFs (Figure 1E).

**FIGURE 2:**
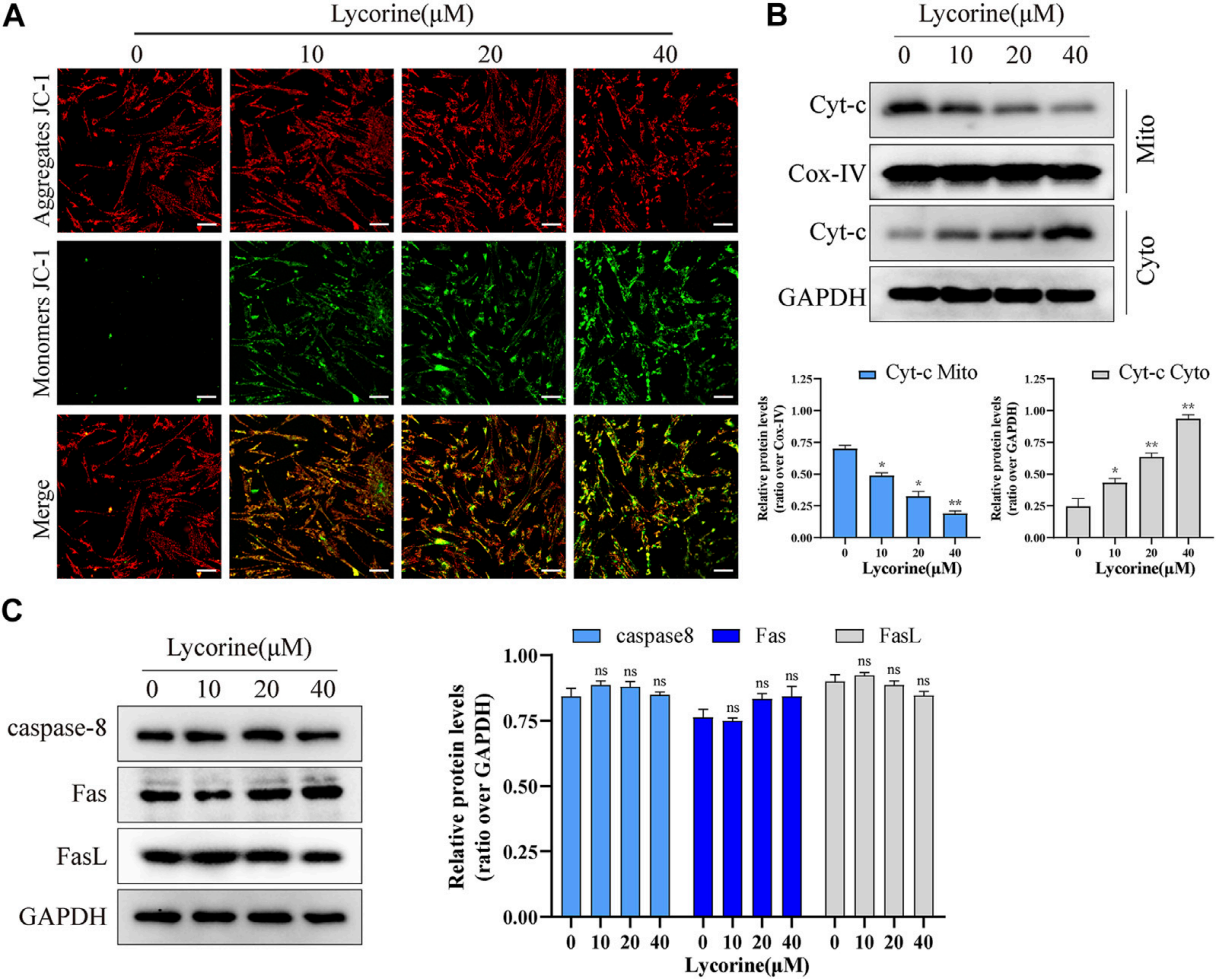
Lycorine induced mitochondrial apoptosis of HSFs. **(A)** HSFs were treated with vehicle or lycorine for 24 h, and the mitochondrial membrane potential (MMP) was analyzed by fluorescent imaging after staining the cells with JC-1 (*n* = 3). Scale bar: 200 μM. **(B)** At 24 h post lycorine treatment, the relative levels of Cytochrome C (cyt-C) in both the cytoplasm and mitochondria were examined by Western blot (*n* = 3). **(C)** At 24 h post lycorine treatment, the relative levels of caspase 8, Fas, Fasl to the control GAPDH expression in NFs were examined by Western blot (*n* = 3). Data are mean ± standard error of the mean.

To further confirm that lycorine acted through the intrinsic apoptotic pathway, the mitochondrial membrane potential (MMP) of individual groups of cells was measured by fluorescent imaging after staining the cells with JC-1. As shown in Figure 2A, compared with the cells treated with vehicle alone, treatment with different doses of lycorine reduced the contents of red mitochondrial aggregates, but enhanced green monomer signals in HSFs. Subsequently, treatment with varying doses of lycorine dose-dependently decreased the relative levels of mitochondrial cytochrome C, but increased cytoplasm cytochrome C, indicating that the decreased MMP by lycorine treatment promoted the release of cytochrome C from the mitochondria into the cytoplasm in HSFs (Figure 2B). However, there was no obvious alternation in the relative levels of Fas/FasL and caspase-8 expression in HSFs regardless of lycorine treatment, independent of death-receptor (Figure 2C). Together, lycorine treatment induced the apoptosis of HSFs through the mitochondrial apoptotic pathway.

### Lycorine Reduces the Levels of Fibrosis in Hypertrophic Scar Fibroblasts

**FIGURE 3:**
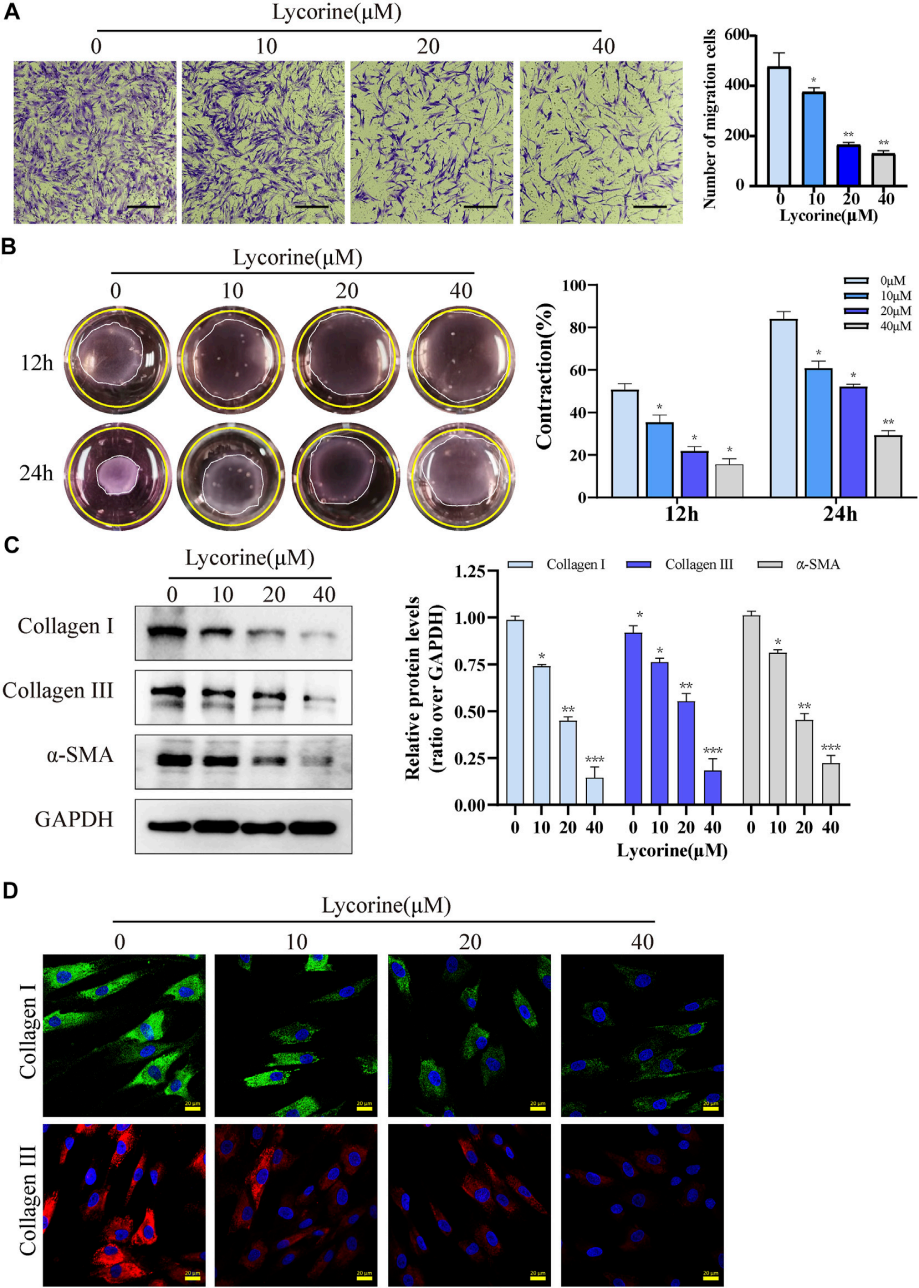
Lycorine inhibits the migration and contraction of HSFs by reducing their production of fibrotic factors. HSFs were treated with vehicle or lycorine at indicated concentrations. **(A)** Transwell migration assays indicated that lycorine treatment for 24 h inhibited the migration of HSFs (*n* = 3). Scale bar = 200 mm. **(B)** HSFs were mixed with rat tail collagen, and the collagen gesl were imaged at 12 and 24 h later (*n* = 3). **(C)** At 24 h post lycorine treatment, the relative levels of a-smooth muscle actin (a-SMA), collagen I, and collagen III expression were determined by Western blot (*n* = 3) and **(D)** the levels of collagen I, collagen III expression were analyzed by immunofluorescence (*n* = 4), Scale bar = 20 μM. Data are mean ± standard error of the mean. *p < 0.05, **p < 0.01, ***p < 0.001.

Besides hyperproliferation, the migration and contraction characteristics of myofibroblasts are also the main pathological features of HS. First, the characteristics of myofibroblasts in primary HSFs were examined. The relative levels of α-SMA, type I collagen and type III collagen in HSFs were significantly higher than that of NFs (Supplementary Figure S1A). Transwell migration assays unveiled that lycorine treatment significantly reduced the number of migrated cells (Figure 3A). Similarly, collagen gel contraction assays exhibited that lycorine treatment attenuated the contractile ability of HSFs (Figure3B).

Next, the anti-fibrosis ability of lycorine was examined. Both Western blot and immunofluorescence revealed that lycorine treatment dose-dependently decreased the relative levels of Collagen I and III, α-SMA expression in HSFs (Figures 3C,D). Collectively, these data indicated that lycorine treatment inhibited the profibrotic activity of HSFs.

### Lycorine Stimulates High Levels of Reactive Oxygen Species Production, Leading to HSF Apoptosis

**FIGURE 4:**
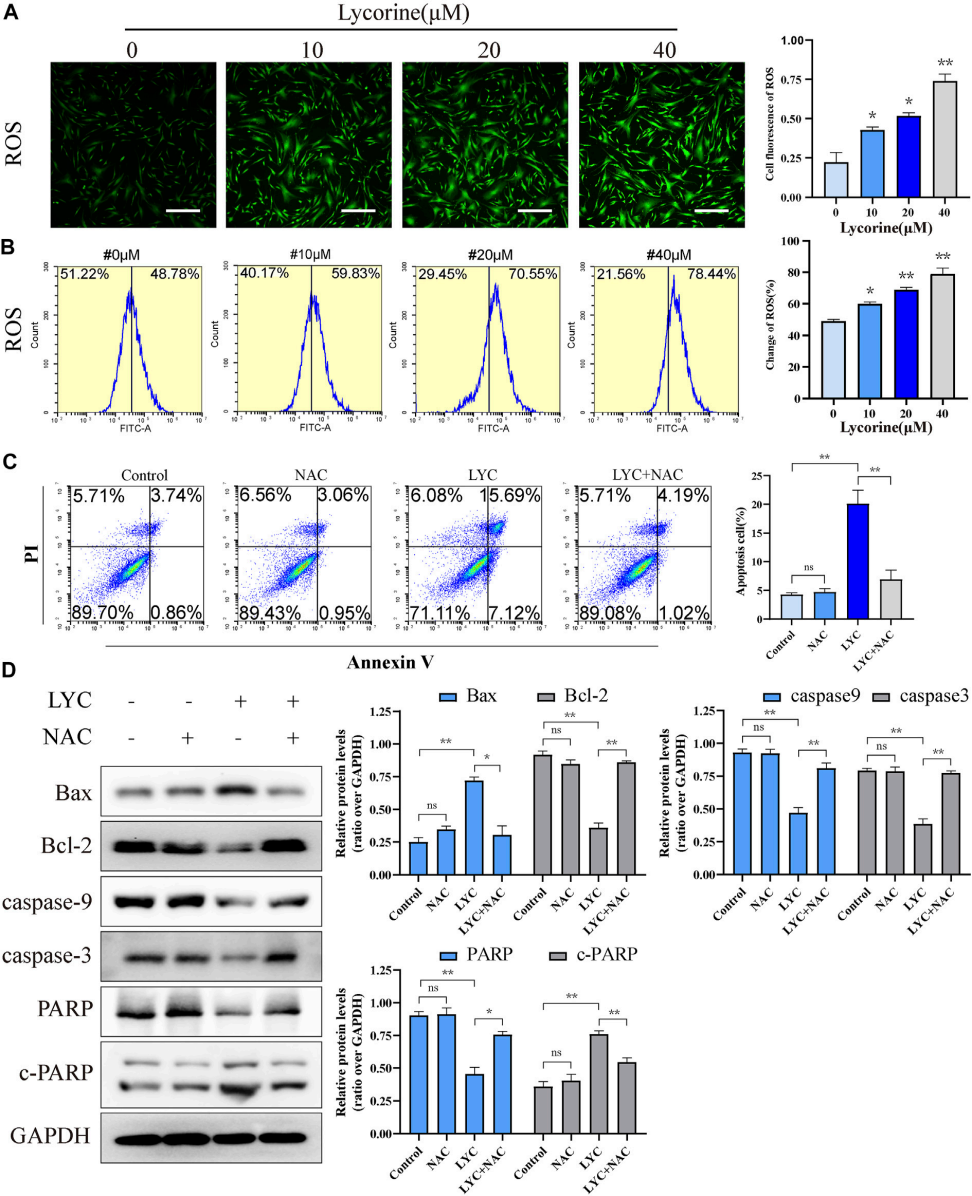
Lycorine stimulates high levels of reactive oxygen species (ROS) production, leading to the apoptosis of HSFs. (A–B) HSFs were treated with vehicle or lycorine at indicated concentrations. The levels of intracellular ROS were analyzed by **(A)** immunofluorescence (*n* = 4, Scale bar = 400 μM) and **(B)** flow cytometry (*n* = 4). **(C)**Flow cytometric analysis of the percentages of apoptotic HSFs following treatment with vehicle (control) or 40 μM lycorine in the presence or absence of 100 μM NAC for 24 h (*n* = 4). **(D)** The relative levels of a-smooth muscle actin (a-SMA), collagen I, and collagen III expression in the different groups of cells were analyzed by Western blot (*n* = 3). Data are mean ± standard error of the mean. *p < 0.05, **p < 0.01, ***p < 0.001.

High levels of ROS can induce oxidative stress and cell apoptosis. To understanding the pharmacological action of lycorine, the effect of lycorine on ROS production was tested by fluorescent microscopy and flow cytometry. As shown in Figure 4A, treatment with varying doses of lycorine increased ROS levels in HSFs cells. A similar pattern of ROS levels was detected by flow cytometry (Figure 4B).

**FIGURE 5:**
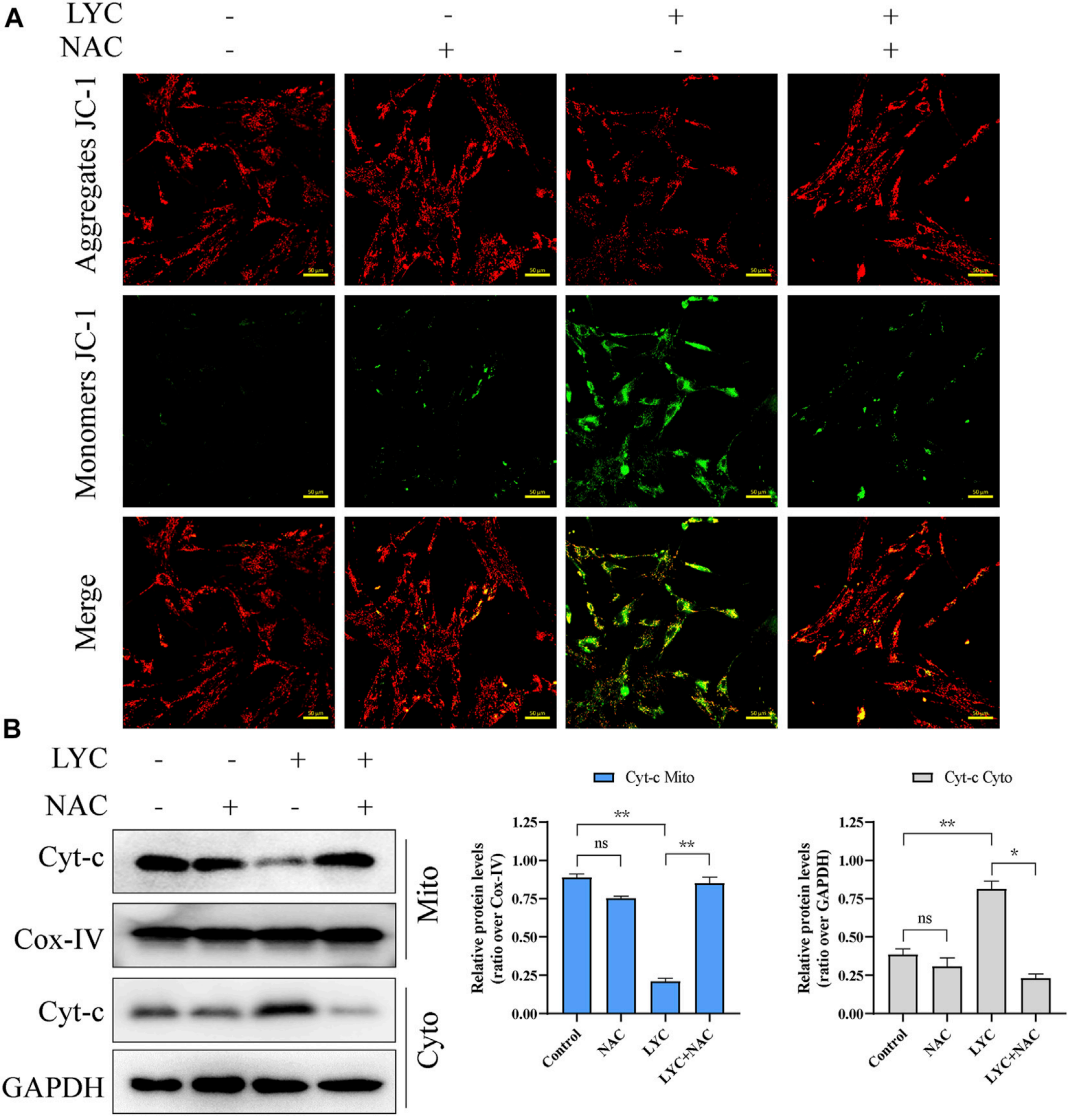
High levels of ROS induced by lycorine alters the mitochondrial membrane potential (MMP) and cytochrome C (cyt-C) translocation. HSFs were treated with vehicle (control) or 40 μM lycorine in the presence or absence of 100 μM NAC for 24 h. **(A)** The MMP was analyzed by fluorescence microscopy after staining with JC-1 (*n* = 3). Scale bar: 400 μM. **(B)** The levels of Cytochrome C (cyt-C) in the both cytoplasm and mitochondria were examined by Western blot (*n* = 3). Data are mean ± standard error of the mean. *p < 0.05, **p < 0.01, ***p < 0.001.

To determine the role of ROS in the lycorine-triggered HSF apoptosis, HSFs were treated with vehicle or lycorine in the presence or absence of 100 μM N-Acetylcysteine (NAC, a ROS inhibitor) (Ding et al., 2021) and the frequency of apoptotic HSFs was quantified by flow cytometry. First, the use of NAC alone or in combination with lycorine both can reduce the ROS level (Supplementary Figure S1B). Treatment with NAC alone did not alter the frequency of spontaneously apoptotic HSFs (Figure 4C). While treatment with 40 µM lycorine triggered 22.81% of apoptotic HSFs treatment with the same dose of lycorine, together with NAC, significantly mitigated the percentages of apoptotic HSFs to 5.21%. Furthermore, compared with lycorine alone, treatment with NAC decreased the levels of Bax and c-PARP, but increased Bcl-2, Caspase9, Caspase3, PARP expression in the lycorine-treated HSFs (Figure 4D). In addition, treatment with NAC also mitigated the decrease in the MMP and attenuated the translocation of Cyto-C from the mitochondria to the cytoplasm in HSFs (Figures 5A,B). Thus, lycorine stimulated high levels of ROS production, leading to HSF apoptosis.

### Lycorine Treatment Alleviates Hypertrophic Scar of Rabbit Ears by Promoting Apoptosis *in vivo*

**FIGURE 6:**
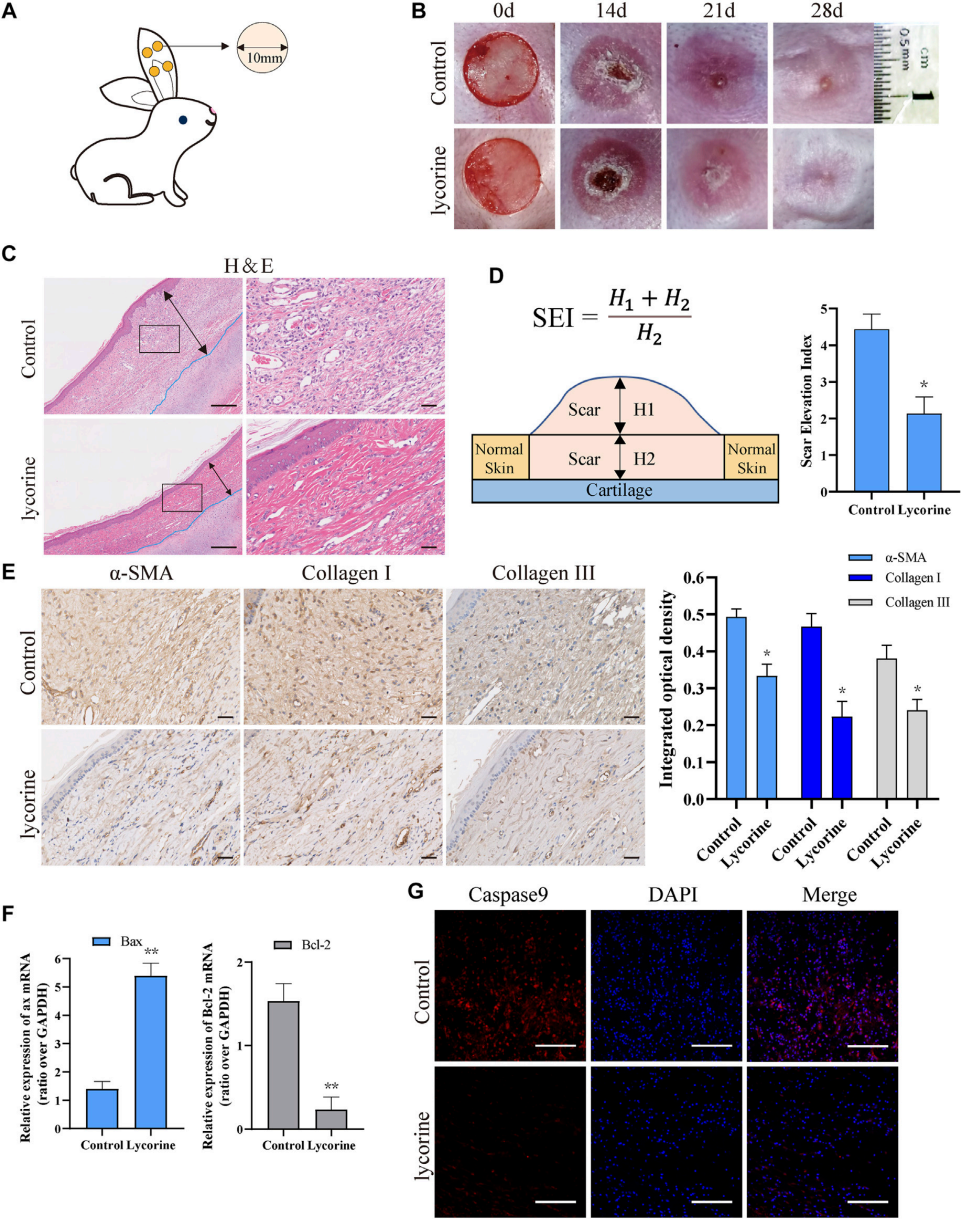
Lycorine treatment attenuates the severity of hypertrophic scar (HS) *in vivo*. At 14 days post surgery in rabbit ears, the rabbits were randomized and injected subcutaneously with vehicle DMSO or lycorine into each rabbit’s ear. **(A)** Schematic diagram of a rabbit ear HS model. **(B)** The wounded ears and scar formation at the indicated time points. **(C)** HE staining of scar tissue sections at 28 days post surgery (*n* = 4). “←→” indicates the scar thickness. Scale bar = 100 mm. **(D)** The analysis of SEI at 28 days post surgery. SEI > 1 indicates HS (*n* = 4). **(E)** Immunohistochemistry analysis of a-smooth muscle actin (a-SMA) , collagen I and Collagen III expression (*n* = 4), Scale bar = 100 mm. **(F)** The relative levels of Bax and Bcl-2 mRNA transcripts in rabbit ear scar tissues at 28 days post surgery (*n* = 4). **(G)** Immunofluorescence analysis of caspase 9 expression in rabbit ear scar tissues (*n* = 4). Data are mean ± standard error of the mean. *p < 0.05, **p < 0.01, ***p < 0.001.

To test the effect of lycorine on HS *in vivo*, a rabbit ear model of HS was established by inducing four 10-mm cylindrical cores (full-thickness skin wounds) on the ventral side of each rabbit ear (Figure 6A). Two weeks later, when the wounds were healed (Figure 5B), the rabbits were randomized and injected subcutaneously with vehicle DMSO (control) or lycorine into the scars, followed by observation up to 28 days post the wounding. There were visible scars at 21 and 28 days post surgery (Figure 6B). While the scars were obviously prominent on the skin surface with darker color and harder texture in the control group of rabbits the scars in the lycorine-treated group appeared smoother, lighter and softer.

Histological analysis revealed that the scar thickness in the control group was significantly higher than that in the Lycorine-treated group at 28 days post surgery. While collagen fibers in the scar tissues of the control group exhibited disorder and rough, the collagen fibers in scar tissues of the lycorine-treated group were arranged in a neat and meticulous way (Figure 6C). The values of scar elevation index (SEI, a measure of the degree of HS) in the scar tissues of the lycorine-treated group were significantly less than that of the control group (Figure 6D).

Immunohistochemical results indicated that the levels of collagen I, collagen III and α-SMA in fibroblasts of scar tissues in the control group were significantly higher than that in the lycorine-treated group (Figure 6E). These suggest that lycorine may attenuate the conversion of fibroblasts to myofibroblast phenotype, contributing to its antifibrotic effect *in vivo*. Further RT-qPCR revealed that lycorine treatment increased the relative levels of Bax mRNA transcripts, but decreased Bcl-2 mRNA transcripts in rabbit ear scar tissues at 28 days post surgery (Figure 6F). Similarly, immunofluorescence displayed that the levels of caspase-9 expression in rabbit ear scar tissue sections from the lycorine-treated group were lower than that in the control group (Figure 6G). Therefore, lycorine treatment attenuated the severity of HS in rabbit ear by promoting fibroblast apoptosis *in vivo*.

## Discussion

The results from this study indicated that lycorine reduced fibrotic activity of HS in two ways. Firstly, lycorine stimulated high levels of ROS production, which promoted the intrinsic apoptosis of HSFs and inhibited myofibroblast proliferation. Secondly, lycorine also inhibited the collagen and α-SMA expression in HSFs, reducing the accumulation of ECM. More importantly, treatment with lycorine significantly mitigated the severity of HS by enhancing the apoptosis of HSFs in a rabbit ear model, demonstrating its pro-apoptotic and anti-fibrosis activities *in vivo*. These novel findings suggest that lycorine may be a promising candidate for development of new therapies for HS.

Lycorine, an alkaloid extracted from *Lycoris*, has a variety of pharmacological effects, including anti-tumor (Wang et al., 2017; Roy et al., 2018), anti-inflammatory (Wang et al., 2018), antibactericidal activities and others (Bendaif et al., 2018), which are associated with triggering cancer cell apoptosis and necroptosis and inhibiting their invasion and metastasis (Luo et al., 2015; Ji et al., 2017; Gao et al., 2021). The data from this study indicated that lycorine treatment selectively decreased the viability of HSFs and triggered their apoptosis, but did not affect NFs, consistent with the results of previous studies (Lamoral-Theys et al., 2009). These findings suggest that HSFs may have unique characteristics and like cancer cells, be sensitive to apoptotic stimulators induced by lycorine.

Apoptosis can be triggered by the intrinsic mitochondrial and extrinsic death receptor pathways. The intrinsic apoptotic pathway is mainly regulated by pro-apoptotic and anti-apoptotic members in the Bcl-2 protein family (Danial, 2007). It is well known that anti-apoptotic Bcl-2 and Bcl-xl can support cell survival by inhibiting the activity of pro-apoptotic Bax and Bak while various endogenous apoptotic stimuli can up-regulate the expression of pro-apoptotic Bax and Bak, which translocate into the mitochondrial outer membrane and polymerize to form membrane channels to promote the release of Cyt-C from the mitochondria, leading to apoptosis (Lopez and Tait, 2015). In fact, lycorine can induce HepG2 cell apoptosis by enhancing cyt-C release form the mitochondria (Liu et al., 2019). Similarly, we found that lycorine decreased the MMP of HSFs and increased cytoplasm cyt-C in HSFs besides the increased frequency of apoptotic HSFs. In the cytoplasm, the Cyt-C, together with caspases (mainly caspase 9) formed apoptotic bodies to drive a cascade of downstream caspases (mainly caspase 3), leading to cell apoptosis. Consistently, lycorine treatment decreased the relative levels of caspse-3, caspase-9 and PARP in HSFs, which may reflect an increase in the degradation of Caspse9, Caspase3 and PARP, similar to that in oral squamous cell carcinoma HSC-3 cells (Li et al., 2021). However, we did not find that lycorine treatment significantly modulated the levels of caspase-8, Fas and FasL expression in HSFs. These data were in disagreement with previous observations that lycorine induces the apoptosis of human osteosarcoma and breast cancer cells through the exogenous death receptor pathway (Fritsch et al., 2019; Newton et al., 2019). Therefore, lycorine can trigger the apoptosis of varying types of pathogenic cells through different apoptotic pathways, dependent on the cell origination and state.

HSFs are different from NFs as HSFs have the characteristics of myofibroblast cells with high levels of α-SMA expression (Tutuianu et al., 2019). In this study, we extracted primary HSFs from human scar tissues and found that they were α-SMA (+) and had potent contractility, contributing to HS formation. In addition, the extracted primary HSFs, like myofibroblasts, also had aberrant migration and proliferation abilities and produced high levels of collagens (type III collagen was dominant in the early stage and type I collagen in the late stage) that might be deposited in the ECM, leading to HS formation. Because lycorine also has anti-fibrosis potential we investigated the effect of lycorine on major fibrosis factors. The data unveiled that lycorine treatment inhibited the migration and contraction of HSF, the expression of α-SMA, and the production of collagen I and collagen III, extending previous observation in colorectal cancer cells (Gao et al., 2021). Therefore, our data support the notion that lycorine can attenuate myofibroblast phenotype.

Our data indicated that lycorine modulated mitochondrial homeostasis and induced the apoptosis of HSFs. Evidently, lycorine treatment decreased the MMP, which is an important indicator of oxidative stress (Sinha et al., 2013; Demine et al., 2019). Given that ROS levels are determinants of oxidative stress-related apoptosis or the changes in the MMP (Cadenas, 2018; Su et al., 2019), we examined the effect of lycorine on ROS production in HSFs. Similar to a previous report (Li et al., 2021), we found that lycorine treatment stimulated high levels of ROS production in HSFs and produced high levels of ROS appeared to be responsible for the pro-apoptotic and anti-fibrotic activities of lycorine. Supportively, treatment with NAC, a ROS scavenger (Sassetti et al., 2021), alone did not affect the apoptosis of HSFs, but significantly mitigated or abrogated the pro-apoptotic and anti-fibrotic effects of lycorine in HSFs by increasing the MMP, reducing cytoplasm cyt-C translocation and apoptosis.

Finally, to explore the effect of lycorine on HS *in vivo* (Li et al., 2020), we established the most classical rabbit ear model of HS. We found that lycorine treatment mitigated the severity of HS by enhancing HSF apoptosis in a rabbit ear model of HS. Therefore, high levels of ROS induced by lycorine may be critical for its pharmacological actions in inhibiting HS formation.

## Conclusion

**FIGURE 7:**
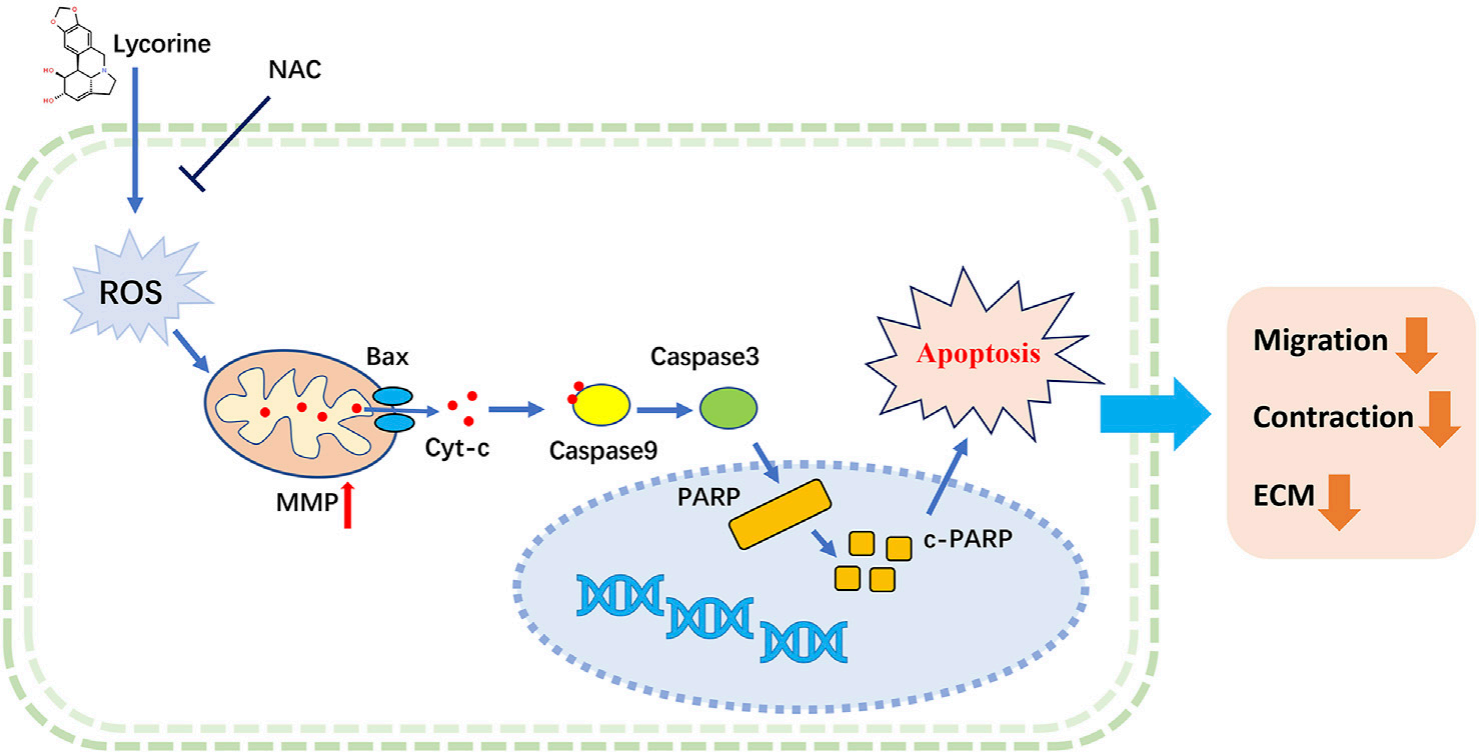
A Schematic diagram for ROS-mediated mitochondrial apoptosis of lycorine in hypertrophic scar fibroblasts (HSFs).

In summary, our study indicated that lycorine stimulated the production of high levels of ROS to induce the apoptosis of HSFs through the intrinsic mitochondrial pathway, and reduce their migration and collagen contraction ability, as well as the accumulation of ECM (Figure 7). Similarly, lycorine treatment mitigated the severity of HS *in vivo* by enhancing the apoptosis of HSFs. Therefore, lycorine may be a promising drug for treatment of HS.

## Data Availability

The original contributions presented in the study are included in the article/Supplementary Material, further inquiries can be directed to the corresponding authors.
