# Supplementary figures and images for: Lycorine Inhibits Hypertrophic Scar Formation by Inducing ROS-Mediated Apoptosis
Source: Front Bioeng Biotechnol. 2022 May 24;10:892015. doi: 10.3389/fbioe.2022.892015 (PMC9171077; doi:10.3389/fbioe.2022.892015)

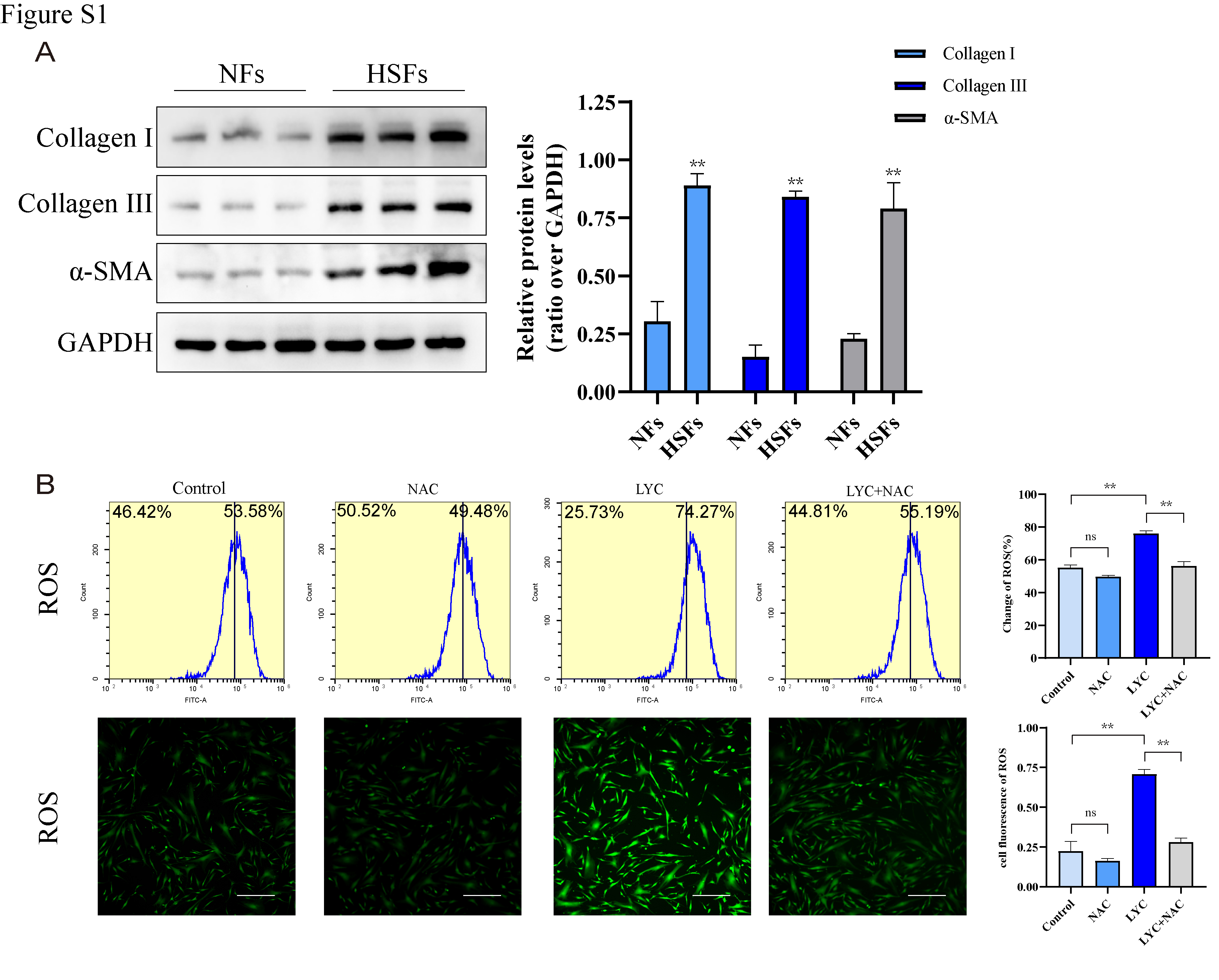

Supplement: Supplementary file 1 [file Image1.JPEG]
